# Social determinants of health and HIV Pre-Exposure Prophylaxis (PrEP) interest and use among young Black and Latinx sexual minority men

**DOI:** 10.1371/journal.pone.0267031

**Published:** 2022-04-15

**Authors:** Tyler M Andriano, Julia Arnsten, Viraj V. Patel

**Affiliations:** 1 Albert Einstein College of Medicine, Bronx, New York, United States of America; 2 Division of General Internal Medicine, Albert Einstein College of Medicine, Montefiore Medical Center, Bronx, New York, United States of America; David Geffen School of Medicine at UCLA, UNITED STATES

## Abstract

Young Black and Latinx sexual minority men (YBLSMM) have low use of HIV pre-exposure prophylaxis (PrEP), despite high rates of new HIV diagnosis. While unmet social determinants of health (SDOH) have been associated with low uptake of preventive health services, this association is unknown for PrEP. To understand the relationship between SDOH and PrEP adoption in this population, we analyzed data from an online survey of HIV-negative YBLSMM aged 18–29 in New York City (n = 143). Participants completed a 17-item SDOH needs scale measuring basic, health/social-services, and economic needs. We used regression models to examine associations of unmet SDOH with outcomes of intention to use PrEP and current PrEP use. Of those not on PrEP (n = 114), 69 (61%) intended to use PrEP. More unmet SDOH needs overall were associated with intention to use PrEP (OR 1.4; 95% CI 1.1, 2.0), as were more unmet basic needs (OR 1.7; 95% CI 1.1, 2.5) and more unmet economic needs (OR 1.3; 95% CI 1.0, 1.7). Unmet SDOH needs were not associated with current PrEP use. Findings suggest that intention to use PrEP among YBLSMM is a likely marker of unmet SDOH needs, as YBLSMM with unmet needs may have limited resources to support moving from intention to actual use. Future research should evaluate programs engaging YBLSMM intending to use PrEP with interventions to screen for and address SDOH.

## Introduction

From 2014 to 2018, new annual HIV diagnoses in the United States decreased from 40,836 to approximately 37,000, with almost all at-risk populations experiencing declines. However, young Black and Latinx sexual minority men (YBLSMM) continue to experience a disproportionate burden of new HIV infections. In 13–24 year old sexual minority men (SMM), Black/African American and Hispanic/Latinx accounted for the highest percentage of new HIV diagnoses (52% and 27%, respectively) [1]. Pre-exposure prophylaxis (PrEP) with oral tenofovir disoproxil fumarate and emtricitabine (TDF/FTC) is highly effective at preventing new HIV infections [2]. However, data of SMM in the United States supports unequal PrEP access, uptake, and persistence across different ages, races, and ethnicities [3–9]. Of 78,360 PrEP users in 2016, only 11.2% were Black/African American and 13.1% were Hispanic [10]. Reasons identified for low PrEP uptake among YBLSMM include concerns about side effects, perceived high cost, limited access, and stigma [11–14].

Social determinants may also contribute to low PrEP uptake among YBLSMM. The uptake of biomedical prevention tools and health services in general has been shown to be impacted by unmet social determinants of health (SDOH) needs [15]. These include basic needs (e.g., food, shelter, water), health/social service needs (e.g., healthcare), and economic needs (e.g., money for savings). Socioeconomic factors identified to affect PrEP uptake in the general population included lack of insurance, difficult access to transportation, and inflexible work situations [16, 17]. Individuals with more unmet SDOH needs may have other priorities that divert their focus from obtaining preventative health services [18].

Young SMM have substantial financial hardship and SDOH needs, which may impact their health service utilization [15]. However there is a paucity of information about SDOH needs among HIV-negative YBLSMM. Additionally, there is little known about associations of SDOH needs with PrEP uptake in YBLSMM. Our research investigated associations of SDOH needs with both intention to use and current use of PrEP in a sample of YBLSMM. We hypothesized that more unmet SDOH needs would be associated with lower proportions of both intention to use and current use of PrEP.

## Methods

### Study design and setting

We performed a secondary analysis of data from the Empowering with PrEP (E-PrEP) study, a cluster-randomized controlled trial of a peer-based social network intervention to increase PrEP adoption among YBLSMM in New York City [19]. The intervention took place in 2017 completely online, with follow-up surveys completed at baseline, six weeks, and twelve weeks. The six-week assessment included questions about SDOH needs. The Albert Einstein College of Medicine Institutional Review Board approved the E-PrEP study and this analysis.

### Participants

Inclusion criteria were: 1) identification as or assigned male at birth (e.g., participants may identify as transgender, gender non-conforming/non-binary, queer, etc.), 2) Black or Latinx race/ethnicity, 3) age 18–29 years, 4) fluency in English, 5) HIV-uninfected or unknown by self-report, 6) residence in New York City, 7) current Facebook or Instagram account, 8) history of insertive or receptive anal sex with a male partner *and* one or more of the following in the past twelve months: condomless anal intercourse, anal sex with more than three men, sexually transmitted infection, or a sex partner at least 10 years older. Of the 152 participants enrolled in the E-PrEP study, this analysis includes data from all participants who completed the six-week follow-up assessment (n = 143 [94%]).

### Measures

We collected participant age, gender identity, borough of residence, race/ethnicity, sexual orientation, education level, income, employment, living situation, health insurance status, and type of health insurance.

The primary independent variable was SDOH needs measured by a 17-item scale (Cronbach’s Alpha = 0.96) previously validated among a sample of YBLSMM in New York City [15]. The SDOH needs scale asked participants whether they and their family currently had adequate resources to meet specific needs right now, such as food for two meals per day, a house or apartment, medical care, or money to save. Items in the scale were grouped into three subscales: 11-items on basic needs (e.g., food, shelter, water), 2-items on health/social service needs (e.g., access to medical care), and 4-items on economic needs (e.g., money to save). Each item had 6 answer options: “never,” “rarely,” “less than half of the time,” “about half of the time,” “more than half of the time,” or “always” (S1 Table). The mean of responses to all 17 items was computed to determine the full score. The mean responses to items for each subscale (basic needs, health/social service needs, and economic needs) were also computed. Mean scores ranged from 1 (never had enough resources for indicated items) to 6 (always had enough resources for indicated items), with lower scores indicating more unmet SDOH needs.

The primary outcome was intention to use PrEP, measured by the following question: “PrEP is currently available with a prescription from your doctor and research has shown that a majority of insurance companies cover most or all of the costs of PrEP. Do you plan to begin PrEP in the next 30 days?” (yes/no). The secondary outcome was current use of PrEP, measured by the following question: “Are you currently taking PrEP for HIV prevention?” (yes/no).

### Statistical analysis

We first calculated frequencies of sociodemographic characteristics of the sample. We then calculated mean scores for the full SDOH needs scale and for each subscale (range from 1 to 6), and used Wilcoxon signed-rank tests to examine differences between subscale scores. To aid in interpretation and exploration of the data, we also dichotomized the mean score into met SDOH needs (score = 6) and unmet SDOH needs (score <6). Finally, we performed bivariable and multivariable logistic regression to determine associations between each outcome (intention to use PrEP and current PrEP use) and SDOH needs. We controlled for age and race/ethnicity in all multivariable models and report adjusted odds ratios (aOR) and 95% confidence intervals (CI). We also conducted a sensitivity analysis controlling for intervention arm in addition to age and race/ethnicity. We inspected variables in the models for collinearity and checked model fit using Hosmer and Lemeshow goodness of fit test. IBM SPSS Statistics, version 25, was used for all analyses.

## Results

### Participant characteristics

The mean age of the 143 participants was 24 years and the majority identified their current gender as male (87%). Over half (51%) of participants lived in the Bronx. All participants identified as either Latinx/Hispanic (37%) or Non-Hispanic Black (63%), and most identified as gay/homosexual (76%). Many participants (37%) had a high school education or less, 27% were unemployed, and a minority (15%) reported no income. Participants reported a range of living situations, including living with their parents or family (41%) and no place to live (6%). Among participants with health insurance (79%), 53% had Medicaid (Table 1).

**Table 1:** Sociodemographic characteristics (n = 143).

Characteristic		n = 143	
**Age** (Mean, SD)		24 (3)	%
**Gender identity**			
	Male	124	87%
	Female/transgender female	8	6%
	Transgender male	2	1%
	Gender non-conforming/ non-binary	2	1%
	Queer	7	5%
**Borough of residence**			
	Bronx	73	51%
	Brooklyn	31	22%
	Manhattan	27	19%
	Queens	10	7%
	Staten Island	2	1%
**Race/ethnicity**			
	Latinx/Hispanic	53	37%
	Non-Hispanic Black	90	63%
**Sexual orientation**			
	Gay/homosexual	109	76%
	Queer	13	9%
	Bisexual	15	11%
	Heterosexual/straight	2	1%
	Other	4	3%
**Educational level**			
	High school or less	53	37%
	Some college	64	45%
	College or more	26	18%
**Employment** ^a^			
	Full time	53	37%
	Part time	32	22%
	Unemployed	39	27%
	Disabled	5	4%
	Student	23	16%
**Income**			
	None	22	15%
	Less than $10,000	36	25%
	$10,000-$19,999	23	16%
	$20,000-$29,999	21	15%
	$30,000-$39,999	25	18%
	$40,000 or more	16	11%
**Living situation** ^b^			
	No place to live	8	6%
	Temporary living situation	18	13%
	Parents/family	58	41%
	Partner/boyfriend/husband	10	7%
	Roommates	36	25%
	Alone	12	8%
	Wife/female partner/girlfriend	1	1%
**Health insurance**			
	Yes	113	79%
	No	27	19%
	Don’t know	3	2%
**Type of health insurance (n = 113)** ^c^			
	Medicaid	60	53%
	Employer/someone else’s employer	36	32%
	Medicare	7	6%
	Some other source	7	6%
	Don’t know/not sure	3	3%

^a^ Results add up to greater than 100% as participants could select more than one category.

^b^Results add up to greater than 100% due to rounding.

^c^Reported percentage is out of n = 113.

### Social determinants of health

The mean score ± SD was 4.7 ± 1.3 for the full SDOH scale, with mean scores ± SD for the three subscales of: 4.9 ± 1.3 (basic needs), 4.2 ± 1.7 (health/social service needs), and 4.2 ± 1.5 (economic needs). Participants had fewer unmet basic needs (higher SDOH scale scores) compared to both health/social service needs (Z = -5.29, *p* < .001) and economic needs (Z = -7.14, *p* < .001).

Analysis of the dichotomized full SDOH needs scale indicated that 127 participants (89%) did not have their SDOH needs met all of the time. Dichotomized results from each subscale demonstrated that 110 participants (77%) did not have basic needs met all of the time, 95 participants (66%) did not have health and social service needs met all of the time, and 110 participants (77%) did not have economic needs met all of the time.

### Association of SDOH needs with PrEP interest and use

Of the 114 participants not using PrEP, 69 (61%) reported intending to use PrEP in the next 30 days. Only 29 participants (20%) were currently using PrEP.

Results from bivariable analyses showed that among participants not using PrEP (n = 114), those intending, compared to those not intending, to take PrEP in the next 30 days (n = 69) had a lower mean score on the full SDOH need scale (4.5 vs. 5.0, *p* = .03), and lower mean scores on all three subscales: basic needs (4.7 vs. 5.3, *p* = .01), health/social service needs (4.1 vs. 4.3, *p* = .55), and economic needs (4.0 vs. 4.6, *p* = .04). In multivariable analyses, these associations remained significant for the full SDOH scale (aOR [95% CI] = 1.4 [1.1–2.0]), the basic needs subscale (aOR [95% CI] = 1.7 [1.1–2.5]), and the economic needs subscale (aOR [95% CI] = 1.3 [1.0–1.7]). There were no significant differences in mean SDOH needs scores between current PrEP use and not currently taking PrEP (Table 2) or between current PrEP use and intending to take PrEP. In sensitivity analysis controlling for intervention arm, findings did not significantly differ for SDOH needs and intention to use PrEP or PrEP use.

**Table 2:** Association of SDOH needs with both intention to use PrEP and current PrEP use.

	Intending to take PrEP in next 30 days (n = 69)	Not intending to take PrEP in next 30 days (n = 45)	Odds of intending to use PrEP	
	Mean (SD)^a^	Mean (SD)	Unadjusted OR (95% CI)	Adjusted OR (95% CI)
**Full scale**	4.5 (1.4)	5.0 (1.0)	1.4 (1.1, 2.0)*	1.4 (1.1, 2.0)*
**Basic needs**	4.7 (1.3)	5.3 (1.0)	1.7 (1.1, 2.5)**	1.7 (1.1, 2.5)*
**Health/social service needs**	4.1 (1.8)	4.3 (1.6)	1.1 (0.8, 1.4)	1.1 (0.8, 1.3)
**Economic needs**	4.0 (1.7)	4.6 (1.3)	1.3 (1.0, 1.7)*	1.3 (1.0, 1.7)*
	**Currently taking PrEP (n = 29)**	**Not currently taking PrEP (n = 114)**	**Odds of using PrEP**	
	Mean (SD)	Mean (SD)	Unadjusted OR (95% CI)	Adjusted OR (95% CI)
**Full Scale**	4.6 (1.3)	4.7 (1.3)	1.1 (0.8, 1.4)	1.0 (0.8, 1.4)
**Basic needs**	4.8 (1.5)	4.9 (1.3)	0.9 (0.8, 1.4)	1.0 (0.8, 1.4)
**Health/social service needs**	4.0 (1.7)	4.2 (1.7)	0.9 (0.8, 1.4)	1.1 (0.8, 1.4)
**Economic needs**	4.2 (1.6)	4.2 (1.6)	1.0 (0.8, 1.3)	1.0 (0.8, 1.3)

*p < .05

**p < .01.

^a^The lower the score, the less often SDOH needs were met (i.e., more unmet SDOH needs).

## Discussion

In one of the first studies exploring the relation of PrEP adoption with SDOH needs among mostly YBLSMM, we found that almost all participants had some unmet needs. We further found that among those not currently using PrEP, intention to use PrEP was associated with greater unmet basic and economic SDOH needs. Surprisingly, we did not observe any associations between PrEP use and SDOH needs.

Individuals with greater unmet SDOH needs are vulnerable to adverse health related outcomes due in part to chronic stressors in daily living [20]. Associations have been seen between chronically stress-inducing life experiences (e.g., socioeconomic disadvantages) and sexually transmitted infection acquisition [21, 22]. Our results suggest that YBLSMM with unmet SDOH needs believe that they would benefit from using PrEP, likely due to concomitant unmet sexual healthcare needs. However, the higher rates of unmet SDOH needs may leave this population without resources to move from intention to action. Research has shown that individuals often forego PrEP due to pressing requirements of daily life, such as employment demands, housing insecurity, or other social hardships [23–25]. The findings from this study underscore the importance of previous recommendations to assess SDOH needs in at risk populations for optimized health intervention benefit. For example, prior research demonstrates that improving housing and neighborhood quality can reduce HIV risk [26]. Additionally, more holistic support may improve PrEP use, as implementation of client centered care addressing social and material needs was associated with high PrEP uptake (79%) in Black men who have sex with men [27, 28]. Such targeted interventions however have not been evaluated in YBLSMM. Examining the impact of SDOH interventions paired with PrEP outreach in YBLSMM may provide valuable insight to addressing discrepancies between intent and use. Nevertheless, our findings help fill a critical void in the literature regarding the impact of SDOH on PrEP adoption in YBLSMM.

Unexpectedly, there were no significant differences in mean SDOH needs scores between current PrEP use and not currently taking PrEP. This may have likely been due to the low PrEP use (n = 29) and relative homogeneity of unmet SDOH needs in this sample, suggesting that people with unmet SDOH needs may need to prioritize other concerns over PrEP. New York City contains a large and robust network of social and medical services, including outreach programs for young SMM and numerous LGBTQ-competent/affirming, Medicaid-accepting or low cost/free medical providers [29]. Among participants in this study, health and social service needs were more likely than other needs to be met all of the time, suggesting that YBLSMM in New York City may be able to access healthcare despite other unmet needs [30]. However, despite access to healthcare, PrEP uptake remained low in our sample, signifying that additional strategies are needed to overcome barriers to PrEP use.

Our findings should be interpreted in the context of this study’s limitations. Given the cross-sectional design, causality cannot be inferred; longitudinal studies would be helpful to understand potential downstream impacts of unmet SDOH needs on PrEP uptake. Participants in this study were all in a similar age range, recruited online, and based in New York City, potentially limiting generalizability to other settings. Given the low PrEP use in this sample, studies with larger YBLSMM samples may help to further elucidate associations between PrEP use and SDOH needs. We conducted this survey in English, and thus findings may not be generalizable to YBLSMM who are not fluent in English, and who may have more unmet SDOH needs.

## Conclusion

Overall, YBLSMM in this study had high unmet SDOH needs and low PrEP uptake. Intention to use PrEP among YBLSMM is a likely marker of unmet SDOH needs, as YBLSMM with unmet needs may have limited resources to move from intention to action. Recognizing this association is crucial for effective care delivery in this population. Future research should evaluate programs engaging YBLSMM intending to use PrEP with interventions to screen for and address SDOH.

## Supporting information

S1 Table Social Determinants of Health (SDOH) need survey items grouped by subscale. (PDF)
