# Supplementary material for: Social determinants of health and HIV Pre-Exposure Prophylaxis (PrEP) interest and use among young Black and Latinx sexual minority men
Source: PLoS One. 2022 Apr 15;17(4):e0267031. doi: 10.1371/journal.pone.0267031 (PMC9012374; doi:10.1371/journal.pone.0267031)
Supplement: S1 Table — (PDF) [file pone.0267031.s001.pdf]

**S1 Table. Social determinants of health (SDOH) need survey items grouped by subscale.**
**Question:** We would like to ask about whether or not you and your family have enough resources to meet the needs of you and your family as a whole as well as the needs of individual family members. Right now, how often are your needs adequately met? **Responses:** 1=Never, 2=Rarely, 3=Less than half of the time (some of the time), 4=About half of the time, 5=More than half of the time (most of the time), 6=Always
**Basic needs**
Food for two meals a day
House or apartment
Money to buy necessities
Enough clothes for you or your family
Heat for your house or apartment
Indoor plumbing or water
Money to pay monthly bills
Dependable transportation
Time to get enough sleep/rest
Furniture for your home or apartment
Telephone or access to a telephone
**Health/social service needs**
Medical care for you or your family
Public assistance (SSI, AFDC, Medicaid, etc.)
**Economic needs**
Good job for yourself or your partner
Money to buy things for yourself
Money for fun/entertainment
Money to save
